# Aryl hydrocarbon receptor regulates programmed cell death in diseases: molecular mechanisms and therapeutic implications

**DOI:** 10.3389/fimmu.2026.1881079

**Published:** 2026-07-28

**Authors:** Yuanyuan Jiang, Zhiyuan Qiang, Like Zhu, Shuo Liang, Yu Huang, Long Xiao, Liwei Zhu, Zhenfang Du, Sheng Qiang

**Affiliations:** 1 Translational Medical Innovation Center, Zhangjiagang TCM Hospital Affiliated to Nanjing University of Chinese Medicine, Zhangjiagang, Jiangsu, China; 2 Southeast University School of Medicine, Nanjing, Jiangsu, China; 3 Department of Obstetrics and Gynecology, Zhangjiagang Hospital Affiliated to Soochow University, Zhangjiagang, Jiangsu, China

**Keywords:** aryl hydrocarbon receptor, cancer, disease treatment, inflammation, programmed cell death

## Abstract

The aryl hydrocarbon receptor (AHR), which is a ligand-activated transcription factor, controls complex transcription programs in a ligand-specific, cell type-specific, and context-specific manner by integrating signals from the environment, diet, microorganisms, and metabolism. Emerging evidence indicates that AHR participates in the regulation of multiple cell death pathways, including apoptosis, necroptosis, autophagy, pyroptosis, and ferroptosis, playing a crucial role in influencing the pathogenesis of diseases. Originally identified as a sensor for environmental toxins, AHR is now recognized for its interactions with both endogenous and microbial ligands, allowing it to orchestrate complex biological processes. This review synthesizes current knowledge on the structural dynamics, ligand diversity, and activation mechanisms of AHR, while highlighting its dual roles in programmed cell death (PCD). Additionally, we discuss the significance of AHR-regulated cell death in inflammatory diseases, neurological disorders, respiratory diseases, and cancer, highlighting its potential as a therapeutic target for various human conditions.

## 1 Introduction

Programmed cell death (PCD) constitutes one of the core processes through which living organisms maintain homeostasis (1). Its proper activation is crucial for clearing abnormal cells, regulating tissue development, and responding to various physiological and pathological contexts, such as infection (1). However, when cell death pathways become dysregulated or overactivated, they cannot only directly cause tissue damage but also trigger or amplify inflammatory responses through mechanisms including the release of damage-associated molecular patterns (DAMPs) (2). This, in turn, contributes to the initiation and progression of numerous diseases, including autoimmune disorders, neurodegenerative diseases, and malignant tumors (3). Therefore, an in-depth dissection of the key molecules and networks that regulate cell death is significantly important for elucidating the pathological mechanisms of related diseases and developing novel therapeutic strategies.

The aryl hydrocarbon receptor (AHR) is a ligand-activated transcription factor that has long been regarded as a key sensor for the body’s response to exogenous environmental toxins, such as dioxins and polycyclic aromatic hydrocarbons. It plays a central role in regulating the expression of drug-metabolizing enzymes, including cytochrome P450 family members like CYP1A1 (4). In addition to this classic function, AHR is now known to be expressed across a wide range of tissues and cell types, where it integrates environmental, dietary, microbial, and metabolic signals to regulate multiple physiological processes, including immune responses, barrier integrity, and cell cycle progression (5, 6). Its ligands include tryptophan metabolites (e.g., kynurenine and FICZ), microbial indole derivatives, and certain dietary compounds, many of which activate AHR with distinct signaling outcomes. Dysregulated activation or suppression of AHR signaling has been closely linked to various pathological conditions, including inflammatory diseases, neurological disorders, and cancer (7). Beyond its well-established role in xenobiotic metabolism, accumulating evidence now indicates that the AHR plays a significant role in the direct regulation of cell death. It can influence the initiation and execution of various PCD programs, including apoptosis, autophagy, and pyroptosis, through both transcription-dependent and transcription-independent mechanisms (8, 9). AHR also serves as a major modulator of immunity, directing the differentiation of regulatory T cells, Th17 cells, and innate lymphoid cells, while influencing macrophage polarization and dendritic cell activity (10). The convergence of AHR signaling with inflammatory pathways, particularly NF-κB, STAT3, and NRF2, positions this transcription factor at the nexus of immune regulation and cellular stress responses (11, 12). These studies suggest that AHR may serve as an important molecule connecting environmental signals, cellular homeostasis, and disease outcomes. This review aims to elucidate the mechanisms by which AHR regulates multiple programmed cell death pathways and to discuss the implications of this regulation for disease pathogenesis and therapy. Ultimately, we highlight the potential of targeting AHR as a strategy to improve human health and treatment of diseases.

## 2 AHR signaling pathways

### 2.1 AHR structure and activation mechanism

**Figure 1:**
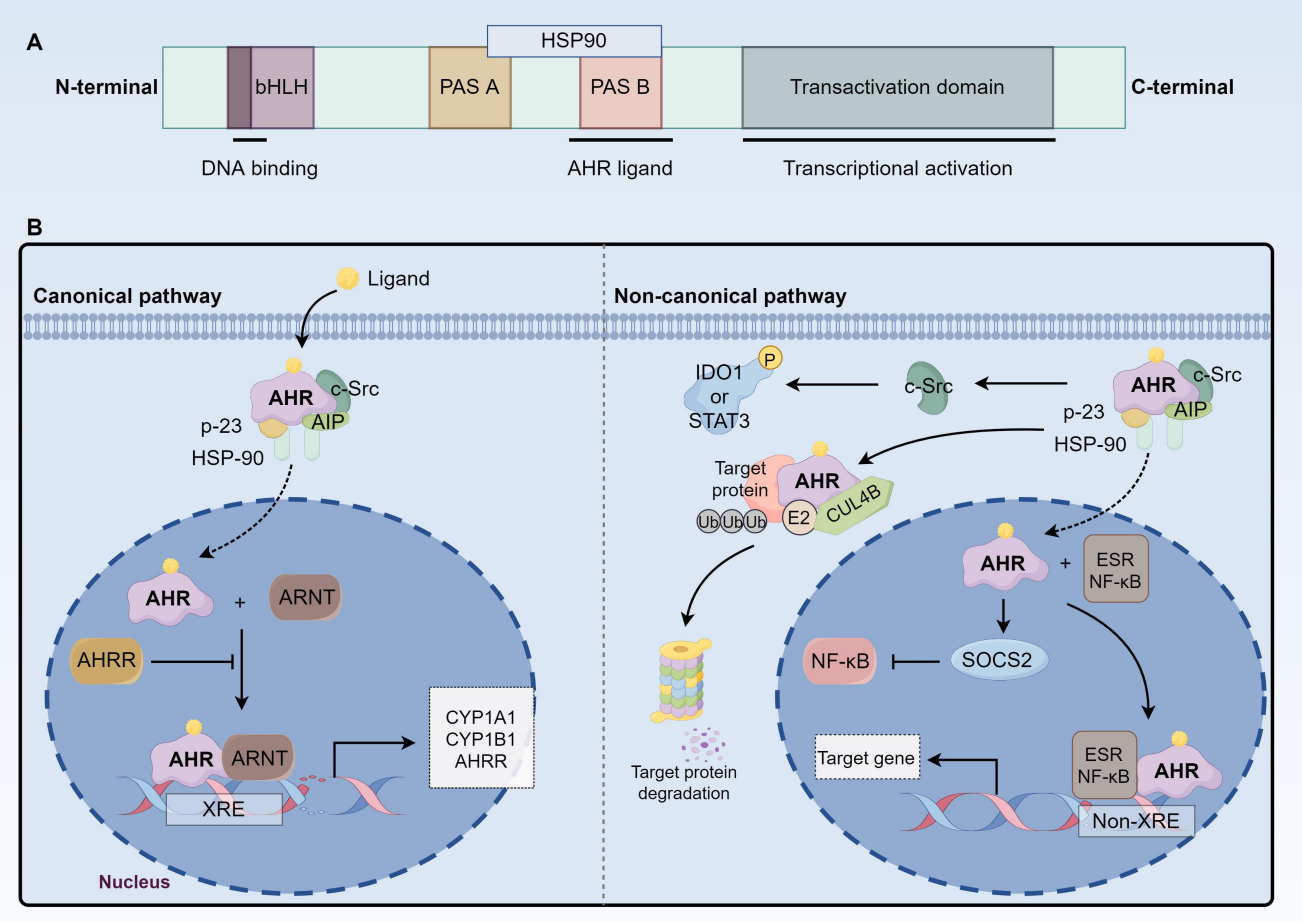
The structure of AHR and its activation mechanism. **(A)** Schematic illustration of the domain organization of AHR. **(B)** Mechanisms of AHR activation. Left (Canonical pathway): Cytosolic AHR is sequestered in a complex with HSP90, p23, AIP, and c-Src. Upon ligand binding, AHR dissociates from this complex, translocates to the nucleus, and heterodimerizes with ARNT. The AHR-ARNT dimer binds to the XRE in the promoters of target genes, driving the transcription of genes such as CYP1A1 and CYP1B1, as well as the negative regulator, AHRR. Right (non-canonical pathway): Upon ligand binding in the cytoplasm, AHR releases c-Src, which mediates the phosphorylation of IDO1 or STAT3. Additionally, AHR can act as a substrate recognition subunit and assemble with CUL4B/E2 to form an E3 ubiquitin ligase complex, thereby inducing ubiquitination and degradation of target proteins. After nuclear translocation, AHR interacts with various transcription factors, such as ESR and NF-κB, to directly or indirectly regulate target gene expression through non-XRE elements. AHR, aryl hydrocarbon receptor; HSP90, heat shock protein 90; AIP, AHR-interacting protein; ARNT, AHR nuclear translocator; XRE, xenobiotic response elements; CYP1A1, cytochrome P450 1A1; CYP1B1, cytochrome P450 1B1; AHRR, AHR repressor; IDO1, indoleamine 2,3-dioxygenase 1; STAT3, signal transducer and activator of transcription 3; CUL4B, cullin 4B; ESR, estrogen receptor; NF-κB, nuclear factor-κB.

AHR is a transcription factor composed of 848 amino acids, and its encoding gene is located on human chromosome 7 (13). This protein comprises the following characteristic domains: (i) a basic helix-loop-helix (bHLH) domain at the N-terminus, which mediates DNA binding; (ii) two central *Per-Arnt-Sim* (PAS) domains, which are involved in ligand binding and interaction with the chaperone heat shock protein 90 (HSP90); and (iii) a C-terminal region containing the transcriptional activation domain responsible for regulating gene expression (**Figure 1A**). Under physiological conditions, the inactivated AHR maintains a high ligand-binding affinity. It co-assembles in the cytoplasm with the HSP90 dimer, c-Src protein kinase, and AHR interacting protein (AIP, also known as X-associated protein 2 or XAP2), along with the cochaperone protein p23 to form a stable complex. Within this complex, HSP90, AIP, and p23 cooperatively maintain the conformation of AHR, preventing its ubiquitination and degradation. This ensures that AHR maintains a high affinity for ligands and remains located in the cytoplasm, while c-Src participates in the initial steps of AHR activation following ligand binding (14, 15).

Upon ligand binding, AHR undergoes conformational changes and translocates into the nucleus. However, it remains unclear whether other components of the AHR chaperone complex co-translocate with AHR into the nuclear compartment (10). In the nucleus, the activated AHR binds to the AHR nuclear translocator (ARNT), also known as hypoxia-inducible factor 1β (HIF-1β), to form a heterodimer. Subsequently, the AHR-ARNT complexes specifically recognize xenobiotic response elements (XREs) containing the canonical 5’-TNGCGTG-3’ sequence. Structural studies reveal that AHR primarily interacts with the 5’-TNGC segment of this motif, while ARNT stabilizes the complex by binding to the adjacent 3’-GTG region (16). This sequence-specific DNA binding event serves as a crucial molecular trigger that activates the transcription of AHR-regulated target genes, including cytochrome P450 enzymes (e.g., CYP1A1 and CYP1B1), which are involved in xenobiotic metabolism, as well as genes associated with various other biological processes (17, 18). Furthermore, the AHR repressor (AHRR), which is also transcriptionally regulated by AHR, can competitively bind to ARNT to form an inhibitory complex, thereby establishing an important negative feedback regulatory mechanism to limit AHR transcriptional activity (19).

In addition to the canonical AHR signaling pathway, AHR can also interact directly or indirectly with other transcription factors, such as the estrogen receptor (ESR) and nuclear factor-κB (NF-κB), to regulate the expression of genes without XRE elements (20). NF-κB signaling is activated by lipopolysaccharide (LPS) and cytokines, such as tumor necrosis factor-α (TNF-α). It mediates the expression of inflammatory factors and genes associated with cell survival (21). Accumulating evidence demonstrates that AHR directly suppresses NF-κB-dependent gene expression and indirectly attenuates NF-κB-driven transcriptional activity by upregulating suppressor of cytokine signaling 2 (SOCS2) (22–24). In recent years, the non-classical pathways of AHR signal transduction have been widely studied, and its regulatory function independent of genomic transcription has also attracted increasing attention. For example, AHR collaborates with cullin 4B (CUL4B) to replace the function of E3 ubiquitin ligase and mediate the ubiquitination and proteasomal degradation of target proteins such as p53 (25), Smad4 (26), and eukaryotic elongation factor 2 kinase (eEF2K) (27). Additionally, through the sequestration of c-Src within its complex, AHR exerts regulatory control over the phosphorylation of c-Src target proteins (28, 29). Studies demonstrate that under LPS restimulation conditions, the release of c-Src from the AHR complex triggers the phosphorylation of indoleamine 2,3-dioxygenase 1 (IDO1), which is essential for tissue homeostasis and immunoregulation (30). Chen et al. recently revealed that AHR-driven signal transducer and activator of transcription 3 (STAT3) hyperphosphorylation promotes IL-10 production, thereby ameliorating lung inflammation through this anti-inflammatory axis (31). This process may be triggered through c-Src-mediated non-genomic signaling (31, 32) (**Figure 1B**).

Notably, emerging evidence indicates that AHR can be activated under ligand-free conditions, although this process may depend on specific stimuli, such as reactive oxygen species (ROS) (33). In conclusion, although AHR has been studied for nearly half a century, the molecular mechanisms by which it mediates the regulation of cellular responses still require further clarification.

### 2.2 AHR ligands

AHR ligands originate from diverse sources, including various categories such as environmental pollutants, host or microbial metabolites, natural compounds, and drugs (34). Environmental pollutants are also classified as exogenous ligands of AHR, with prototypical examples including 2,3,7,8-tetrachlorodibenzo-p-dioxin (TCDD), β-naphthoflavone (β-NF), and the polycyclic aromatic hydrocarbon benzo[a]pyrene (B[a]P). These substances, as powerful agonists of AHR, have been widely studied in the fields of pharmacology and clinical medicine (35). Endogenous AHR ligands encompass both host-derived metabolic products and microbial metabolites from the gut microbiota (36). This classification necessitates the introduction of ‘pro-ligands’—molecular precursors that have limited intrinsic AHR binding capacity or weak agonist effects (37). Through diverse bioconversion pathways, these precursor compounds are metabolically converted into AHR ultimate ligands. The conversion mechanisms may involve enzymatic modifications mediated by host or microbial enzymatic systems, non-enzymatic chemical transformations such as oxidative reactions, condensation processes, and intramolecular rearrangements, or photochemical activation induced by ultraviolet (UV) irradiation (5). A typical example is L-tryptophan, which is an essential dietary amino acid for humans. Although L-tryptophan itself does not activate AHR, it is metabolized in the liver by aspartate aminotransferase or in the intestine by aromatic amino acid aminotransferase into indole-3-pyruvate (I3P), which acts as a ligand that activates AHR (38). Furthermore, L-tryptophan is metabolized to kynurenine (Kyn) by IDO or kynurenine monooxygenase (KMO) (39). While early studies identified Kyn as a low-affinity AHR ligand, recent findings demonstrate that prolonged incubation significantly enhances AHR activation mediated by Kyn (40). This effect is attributed to the formation of Kyn active derivatives, specifically trace extended aromatic condensation products (TEACOPs), which are recognized as potent AHR agonists (40). These data indicate that Kyn likely acts as a precursor to high-affinity AHR ligands. Among microbiota-derived ligands, indole and its derivatives have garnered considerable interest due to their abundance in the intestinal lumen. The bacterial tryptophanase converts L−tryptophan to indole, which is further metabolized by various microbial enzymes into indole−3−acetic acid (IAA), indole−3−propionic acid (IPA), and indole−3−aldehyde (IAld) (41). These metabolites reinforce intestinal barrier function through AHR activation. Microbiota−derived indole metabolites can also reach the central nervous system via the circulation (42). Locally in the brain, tryptophan catabolism through the kynurenine pathway generates metabolites such as kynurenine, which also acts as an endogenous AHR ligand and modulates neural functions (42). Moreover, light exposure, particularly UV radiation, promotes the production of endogenous AHR ligands. In skin tissue, UV radiation photoconverts tryptophan to 6-formylindolo[3,2-b] carbazole (FICZ), which is another high-affinity AHR ligand (43). Similarly, indole-3-carbinol (I3C), derived from cruciferous vegetables, serves as a multifunctional precursor to AHR ligands and agonists. In the gastric environment, it can be converted into 3,3’-diindolylmethane (DIM) and various condensation derivatives (44). These compounds have been confirmed as effective ligands for AHR (45, 46). Additional dietary and host-derived AHR ligands include quercetin, sulforaphane, unconjugated bilirubin, and lipoxin A_4_ (LXA_4_) (47).

Collectively, AHR ligands and agonists primarily originate from three sources: exogenous (e.g., environmental), endogenous (host or microbial metabolic), and dietary. These compounds regulate AHR-dependent signaling cascades, thereby determining cell fate, influencing the tissue microenvironment, and affecting the progression of various human diseases.

## 3 AHR and PCD

PCD, as a highly ordered physiological process, achieves autonomous regulation by responding to internal and external environmental signals (1). This mechanism is essential for fundamental biological processes, including embryonic development, tissue remodeling, and homeostasis control (1). Conversely, PCD dysregulation is strongly implicated in the pathogenesis of diverse human diseases (48, 49). Distinct forms of PCD—including apoptosis, necroptosis, autophagy, pyroptosis, and ferroptosis—determine cell fate through dedicated molecular pathways (50). AHR has been shown to affect multiple types of PCD across various experimental systems, but the extent of this effect differs considerably between cell types and death pathways.

### 3.1 Apoptosis

Apoptosis is the most classic type of PCD, initiated through two major pathways: (a) the extrinsic pathway, mediated by plasma membrane death receptor ligation, and (b) the intrinsic pathway, activated by mitochondrial outer membrane permeabilization (MOMP) (51–53). Ultimately, both pathways converge to activate common effectors, such as caspase-3 and caspase-7, which induce characteristic morphological alterations, including chromatin condensation, cytoplasmic shrinkage, and apoptotic body formation (54). Critically, the preservation of plasma membrane integrity throughout apoptosis prevents inflammatory responses (55).

**Figure 2:**
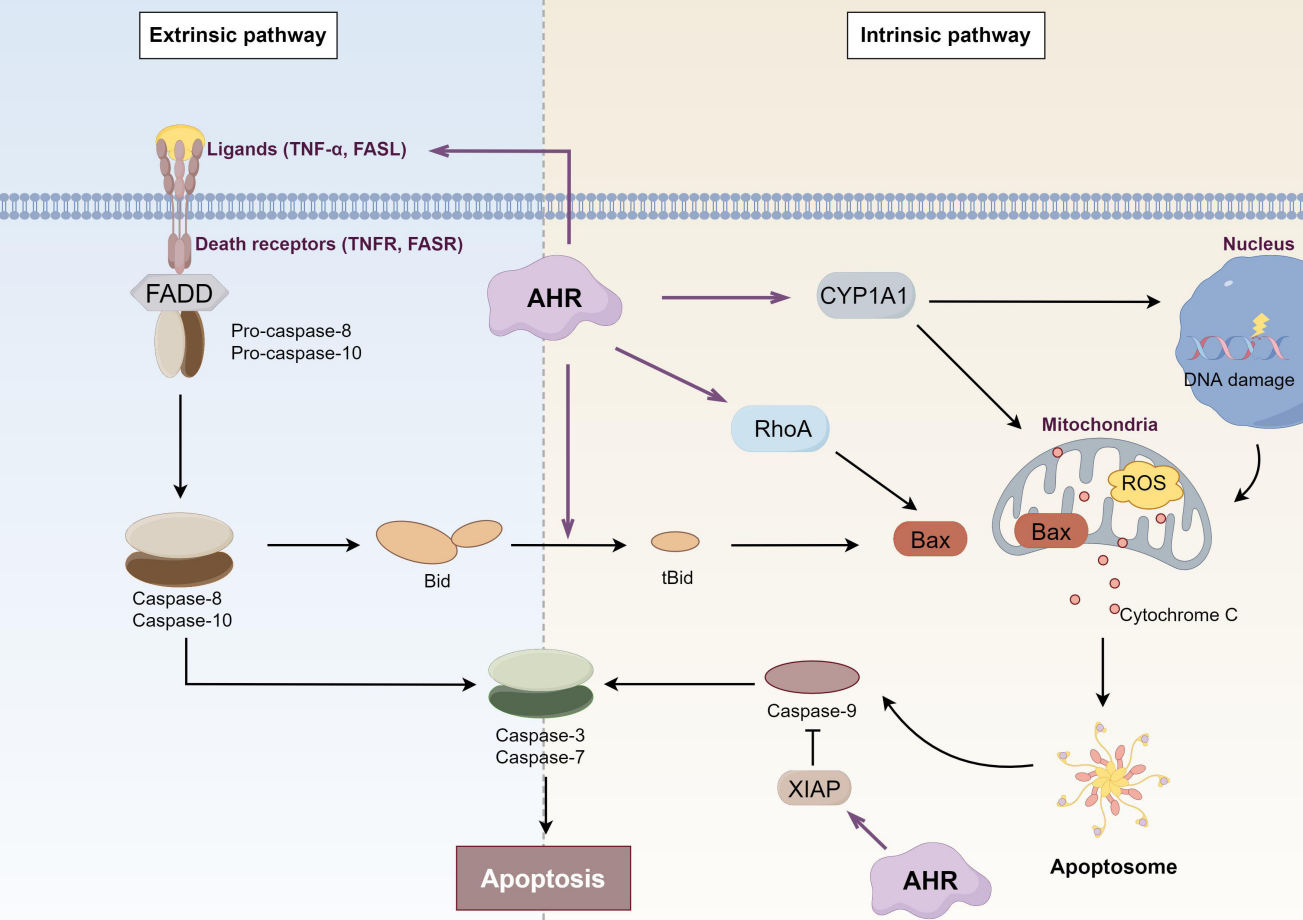
AHR-mediated regulation of apoptotic pathways. Apoptosis is initiated when ligands (TNF-α, FASL) bind to membrane death receptors (TNFR, FASR). This recruits the adaptor protein FADD and converts procaspase-8 and -10 into active caspase-8 and -10. The active caspases either directly cleave executioner caspase-3 and -7 or cleave Bid into its truncated form (tBid). AHR-mediated regulation of apoptotic pathways. Additionally, AHR regulates the intrinsic apoptotic pathway through the following mechanisms: 1) inducing CYP1A1 expression, which leads to nuclear DNA damage and promotes mitochondrial ROS generation; and 2) modulating RhoA to further activate Bax. Activated Bax translocates to the mitochondria and triggers the release of cytochrome c. The released cytochrome c then forms the apoptosome, which activates the conversion of procaspase-9 to caspase-9. AHR also enhances this activation by inhibiting XIAP, a negative regulator of caspase-9. Ultimately, caspase-9 activates caspase-3 and caspase-7, resulting in apoptosis. TNF-α, tumor necrosis factor-α; FASL, Fas ligand; FADD, Fas-associated protein with death domain; Bid, BH3-interacting domain death agonist; AHR, aryl hydrocarbon receptor; CYP1A1, cytochrome P450 1A1; ROS, reactive oxygen species; Bax, Bcl-2-associated X protein; RhoA, Ras homolog gene family member A.

AHR is critically involved in the extrinsic apoptotic pathway, modulating key steps within this cascade (**Figure 2**). The primary extrinsic apoptotic pathway is mediated by caspase-8 activation upon binding of death ligands, such as TNF-α and Fas ligand (FasL), to their cognate receptors (56, 57). Following AHR signaling disruption, this apoptotic cascade is inhibited. Regardless of whether induction occurs via TNF-α or FasL, caspase-8 activation is attenuated (58, 59). These findings demonstrate that AHR positively regulates the extrinsic apoptotic pathway. Intracellular stressors, such as DNA mutations or elevated levels of ROS, activate the intrinsic apoptotic pathway through mitochondrial signaling. In the context of environmental toxicant exposure, AHR activation upregulates CYP1A1, which generates DNA damage and mitochondrial ROS, thereby triggering intrinsic apoptosis (60, 61). Similarly, Melhem et al. demonstrated that the endogenous ligand kynurenine induces ROS−dependent apoptosis through AHR in endothelial cells (62). The Bcl-2-associated X protein (Bax) facilitates MOMP by regulating the formation of pore structures in the outer mitochondrial membrane, serving as a key executor of intrinsic apoptosis (63). BH3-only proteins, such as BH3-interacting domain death agonist (Bid), directly activate Bax. Ligand-activated AHR promotes apoptosis by inducing the Ras homolog gene family member A (RhoA)/Bax signaling axis (64). Additionally, AHR stimulates Bid activation, triggering cytochrome c release from mitochondria and subsequent caspase-9 activation (65).

By contrast, in certain cancer cells, AHR can suppress apoptosis. As a member of the inhibitor of apoptosis protein (IAP) family, the X-linked inhibitor of apoptosis (XIAP) directly inhibits caspase-9 and caspase-3/7 (66). In ovarian cancer cells, elevated AHR expression functions as a competitive endogenous RNA (ceRNA) through its 3’UTR, augmenting XIAP expression and consequently inhibiting cisplatin-induced apoptosis (67). Collectively, AHR exerts opposing effects on apoptosis depending on the cellular context. In normal cells under stress, it predominantly drives pro-apoptotic signals; however, in malignant settings, it shifts toward supporting cell survival by suppressing apoptotic pathways.

### 3.2 Necroptosis

Necroptosis, differing from accidental necrosis and membrane-intact apoptosis, is a caspase-independent programmed cell death pathway characterized by plasma membrane rupture and the subsequent release of intracellular components (68). The molecular mechanism is initiated by the binding of ligands to death receptors and is tightly regulated by the receptor-interacting protein kinase 1 (RIPK1) and RIPK3 cascade. This cascade triggers phosphorylation and oligomerization of mixed lineage kinase domain-like protein (MLKL) (69). Subsequently, oligomerized MLKL translocates to the plasma membrane and assembles into pore-forming complexes, compromising membrane integrity and thereby facilitating the release of intracellular components, notably DAMPs (70). Currently, there is limited research on the regulation of necroptosis by AHR, and its mechanism of action remains controversial. These discrepancies are likely attributable to variations in the tissue environment, ligand identity, and cellular composition. Quan et al. observed that pulmonary exposure to B[a]P—a prototypical environmental AHR agonist—exacerbated inflammatory responses, concomitant with marked alveolar cell necroptosis in murine lung tissue (71). In contrast, in intestinal epithelial cells, dietary I3C activates AHR and enhances RIPK1 ubiquitination, leading to its proteasomal degradation and thereby attenuating necroptotic signaling (72). The latest research further confirms that AHR activation significantly reduces the phosphorylation of key necroptosis mediators (RIPK1, RIPK3, and MLKL) in intestinal epithelial cells (73). The above observations suggest that AHR exerts its influence not through transcriptional regulation of the core necroptotic genes (RIPK1, RIPK3, and MLKL), but rather through upstream checkpoint modulation, with the eventual outcome depending on the balance between ubiquitination and phosphorylation. Collectively, current evidence supports the idea that AHR functions as a contextual modulator rather than as a direct essential component of the necroptotic pathway.

### 3.3 Autophagy

The Nomenclature Committee on Cell Death (NCCD) defines autophagic cell death as a regulated form of cell death characterized by its reliance on the autophagy mechanism, which can only be inhibited by blocking autophagy (74). Nevertheless, autophagy, broadly defined, is an evolutionarily conserved lysosomal degradative pathway that exerts cytoprotective, stress-adaptive, or pro-death effects in a context-dependent manner (75). Three functionally distinct autophagic phenotypes should be clearly distinguished: (a) constitutive cytoprotective autophagy, which sustains basal cellular homeostasis under physiological conditions; (b) stress-triggered autophagy, induced upon nutrient starvation or sublethal cellular injury, which does not inherently drive cell demise; and (c) autophagic cell death, whereby the complete autophagic molecular apparatus directly initiates and mediates cell death signaling cascades (76). The concept of “autophagy” was first described in the mid-19th century as a conserved, lysosome-dependent degradation mechanism (77). Under physiological conditions, autophagy maintains cellular homeostasis and facilitates nutrient recycling through the formation of double-membrane autophagosomes. These vesicles encapsulate damaged organelles, misfolded proteins, or pathogens and subsequently deliver their cargo to lysosomes for degradation. However, under specific pathological stressors, such as oxidative stress or toxin exposure, autophagy can become dysregulated. Sustained or excessive autophagic activation may culminate in autophagic cell death (78).

**Figure 3:**
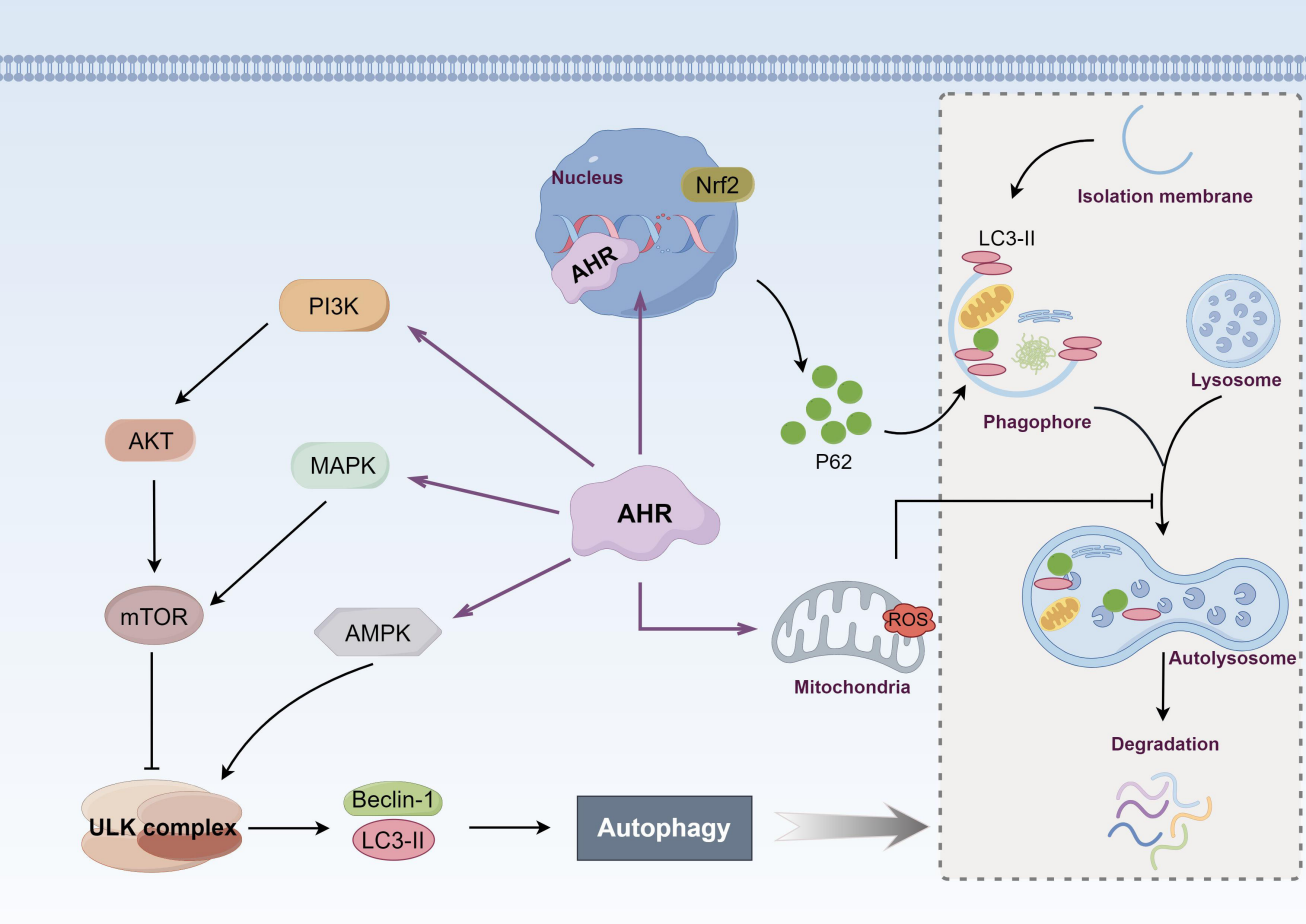
AHR-mediated regulation of autophagy pathways. AHR regulates autophagy through multiple signaling pathways. On one hand, AHR activates the AMPK signaling pathway, which subsequently acts on the ULK complex, a core initiator of autophagy. Activation of the ULK complex upregulates the expression of Beclin-1 and lipidated microtubule-associated protein 1 LC3-II, thereby promoting the formation of the phagophore (also known as the isolation membrane). In addition, AHR modulates cargo receptor p62 to facilitate the recruitment of substrates targeted for degradation into the expanding phagophore. The mature phagophore then fuses with lysosomes to form an autolysosome, ultimately leading to the degradation of the encapsulated contents and the completion of the autophagic flux. On the other hand, AHR can also inhibit the initiation of autophagy by promoting the activation of key signaling pathways such as PI3K/AKT and MAPK, both of which activate mTOR. Furthermore, AHR can interact with the nuclear transcription factor Nrf2, impairing autophagic flux by inducing mitochondrial ROS production. AHR, aryl hydrocarbon receptor; AMPK, AMP-activated protein kinase; ULK, unc-51-like autophagy activating kinase; LC3-II, microtubule-associated protein 1 light chain 3-II; Nrf2, nuclear factor erythroid 2-related factor 2; ROS, reactive oxygen species; MAPK, mitogen-activated protein kinase; AKT, protein kinase B; mTOR, mammalian target of rapamycin.

Accumulating evidence underscores a critical role for AHR in modulating autophagy (**Figure 3**) (79, 80). The autophagy process is coordinated by a network of key regulatory proteins, notably Beclin-1, microtubule-associated protein 1 light chain 3 (LC3), ATG family members, and sequestosome-1 (p62/SQSTM1). Beclin-1 initiates autophagosome nucleation and phagophore formation. LC3-II, a crucial marker associated with autophagosome formation and maturation, facilitates phagophore elongation and closure (81). Functioning as a selective autophagy receptor, p62 recognizes specific substrates, recruits them to the nascent autophagosome via binding to LC3-II, and thereby mediates their degradation (82). In HaCaT keratinocytes, AHR knockout significantly attenuated particulate matter (PM)-induced expression of key autophagy-related genes, including LC3-II and p62 (83). Similarly, Liu et al. demonstrated that suppressing AHR expression in intestinal epithelial cells markedly reduced ATG5, Beclin1, and LC3-II levels, while AHR pathway activation upregulated these proteins (80). Mechanistically, research has found that AHR enhances p62 transcription by promoting the expression of nuclear factor erythroid 2-related factor 2 (Nrf2) (84). Furthermore, AHR has been confirmed to be involved in regulating multiple autophagy-related signaling pathways, including AMP-activated protein kinase (AMPK) (85), mitogen-activated protein kinase (MAPK) (86), and the protein kinase B (AKT)/mammalian target of rapamycin (mTOR) axis (87). Notably, Fu et al. recently demonstrated that environmental toxin-activated AHR triggers substantial mitochondrial ROS release. This ROS surge disrupts autophagic flux, ultimately accelerating cardiomyocyte senescence (88). However, most of the cited studies relied on steady-state measurements of LC3-II, p62, or Beclin-1. While these markers indicate the abundance of autophagosomes, they cannot differentiate between enhanced formation and impaired lysosomal clearance—two processes that have opposing effects on flux. Consequently, while there is strong evidence linking AHR to autophagy, it remains uncertain in many reported systems whether AHR activation augments flux, inhibits clearance, or ultimately drives autophagic cell death. Taken together, these findings indicate that AHR plays a critical yet dual role in regulating autophagy. However, the precise mechanism underlying its direct involvement in autophagy-dependent cell death remains incompletely understood.

### 3.4 Pyroptosis

Pyroptosis is a form of PCD mediated by gasdermin D (GSDMD) cleavage, which forms plasma membrane pores and triggers the release of abundant inflammatory mediators (89). The pyroptotic canonical cascade can be divided into three phases: (a) priming, in which Toll-like receptor (TLR) recognition of ligands such as LPS leads to the activation of the NF-κB pathway and subsequent upregulation of NOD-like receptor (NLR) family pyrin domain-containing 3 (NLRP3) expression; (b) assembly and activation, in which activated NLRP3 recruits the adaptor protein ASC (apoptosis-associated speck-like protein containing a CARD) and pro-caspase-1, forming the NLRP3 inflammasome complex, which leads to caspase−1 autocleavage; and (c) execution, in which active caspase−1 cleaves GSDMD, releasing its N−terminal fragment that forms membrane pores and releases IL−1β and IL−18 (90). NLRP3 inflammasome assembly and activation involve multiple mechanisms, including mitochondrial ROS release, lysosomal destabilization, and efflux of potassium (K^+^) and chloride (Cl^-^) ions (91). Compared to other cell death modalities, pyroptosis significantly exacerbates tissue damage in inflammatory diseases. Therefore, targeted regulation of the pyroptosis pathway is a potential strategy for treating various inflammatory diseases (92, 93).

**Figure 4:**
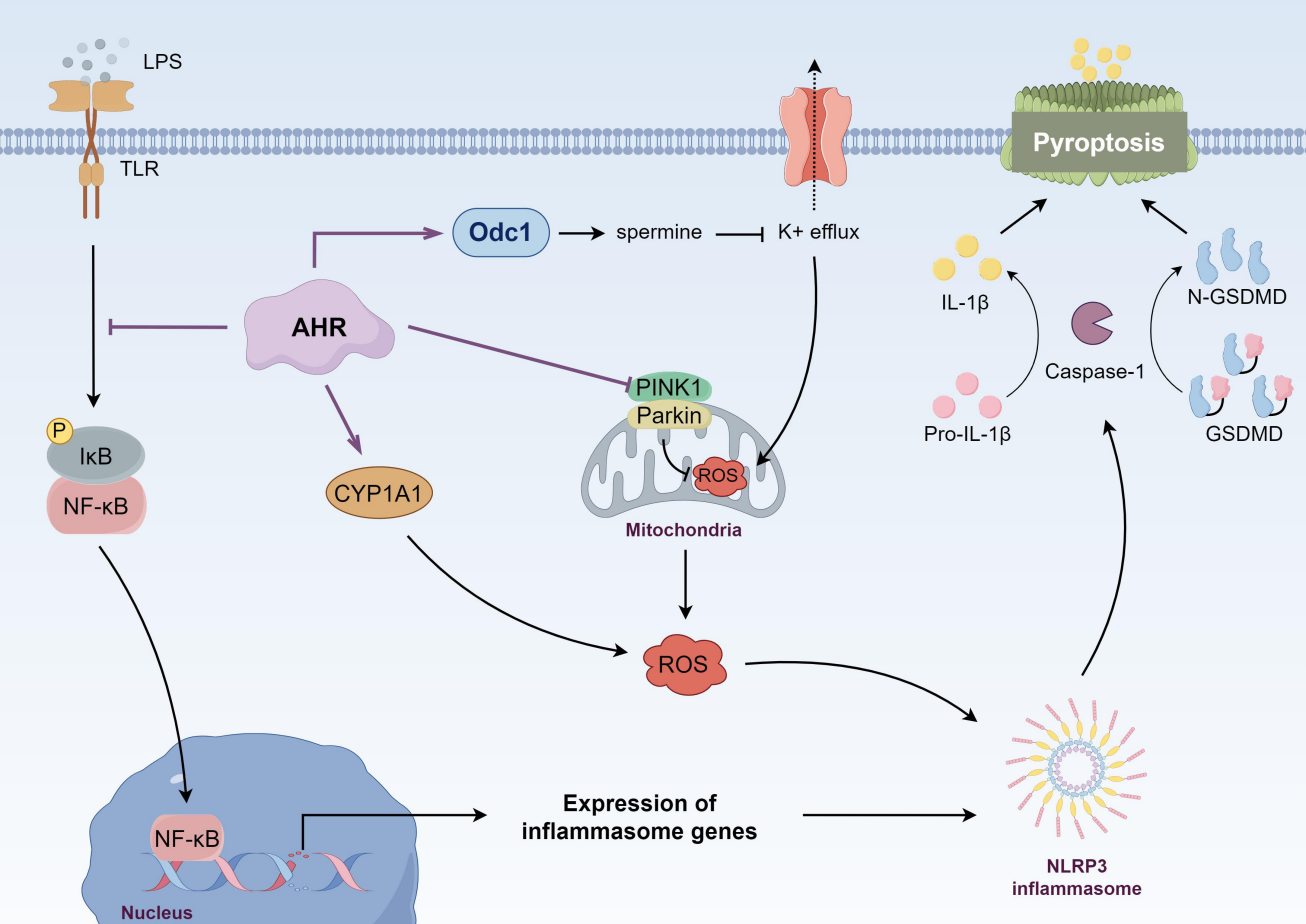
AHR-mediated regulation of pyrotosis pathways. AHR regulates pyroptosis through multiple pathways. On one hand, LPS binding to TLRs induces phosphorylation of IκB, leading to nuclear translocation of NF-κB and subsequent expression of inflammasome-related genes. This process represents the ‘priming’ step of NLRP3 inflammasome activation, which can be inhibited by the AHR. On the other hand, AHR enhances Odc1 expression, leading to spermidine production and subsequent inhibition of K+ efflux. Concurrently, AHR also inhibits the activation of the PINK1/Parkin pathway in mitochondria. These mechanisms collectively reduce mitochondrial ROS generation and inhibit ROS-induced NLRP3 inflammasome activation. However, AHR can also promote ROS production by regulating CYP1A1 expression. Accumulated ROS triggers the activation of the NLRP3 inflammasome, leading to the recruitment and activation of caspase-1. Activated caspase-1 cleaves pro-IL-1β into mature IL-1β and processes GSDMD to release its N-GSDMD. N-GSDMD then inserts into the plasma membrane to form pores, ultimately inducing pyroptosis. AHR, aryl hydrocarbon receptor; LPS, lipopolysaccharide; TLRs, Toll-like receptors; NF-κB, nuclear factor-κB; Odc1, ornithine decarboxylase 1; ROS, reactive oxygen species; CYP1A1, cytochrome P450 1A1; IL-1β, interleukin-1β; GSDMD, Gasdermin D.

Substantial evidence indicates that AHR exhibits context-dependent regulation of pyroptosis (**Figure 4**). Hydroquinone, a benzene metabolite, triggers excessive reactive oxygen species (ROS) generation and endoplasmic reticulum stress via AHR signaling, driving NLRP3 inflammasome-mediated pyroptosis in human lymphocytes (94). Yuan et al. also discovered that exposure to particulate matter (PM_2.5_) induces AHR upregulation in nasal epithelial cells, leading to the transcriptional activation of CYP1A1, increased ROS accumulation, and subsequent NLRP3-dependent pyroptosis, thereby exacerbating allergic rhinitis (95). Additionally, BaP-activated AHR signaling suppresses PINK1/Parkin-mediated mitophagy, promoting mitochondrial permeability transition pore (mPTP) opening and ROS release, which ultimately activates the NLRP3 inflammasome and induces pyroptosis in cardiomyocytes (96). Conversely, AHR activation can also suppress pyroptosis under specific conditions. For instance, in macrophages, AHR activation transcriptionally upregulates ornithine decarboxylase 1 (Odc1), enhancing polyamine biosynthesis to confer cytoprotection (97). The resultant key polyamine, such as spermine, subsequently inhibits the assembly of the NLRP3 inflammasome by suppressing K^+^ efflux, thereby mitigating pyroptosis and intestinal inflammation (97). Similarly, the activation of AHR by spermidine binding inhibits the NF-κB/NLRP3/caspase-1 signaling axis, thereby suppressing chondrocyte pyroptosis and alleviating the progression of osteoarthritis (98). Furthermore, AHR also inhibits pyroptosis in hepatocytes. The long non-coding RNA GAS5 acts as a molecular sponge for miR-684, leading to upregulated AHR expression, which in turn inhibits NLRP3 activation and ultimately blocks the pyroptotic cascade (99). Overall, these findings highlight AHR as a critical yet context-dependent modulator of pyroptosis, with its functions varying across cell types and stimuli. This complex regulatory effect makes AhR a potential therapeutic target for inflammatory pathologies.

### 3.5 Ferroptosis

Ferroptosis is an iron-dependent form of PCD defined by toxic buildup of lipid peroxidation products in phospholipid bilayers (100). Morphologically, cells undergoing ferroptosis exhibit distinct features that distinguish this process from other forms of cell death, including mitochondrial shrinkage, increased density of mitochondrial membranes, and diminished cristae structures (101). Although the key effector proteins executing ferroptosis have not been fully elucidated, current evidence indicates that excessive production of ROS, accumulation of ferrous iron (Fe²^+^), and abnormal buildup of polyunsaturated fatty acids (PUFAs) are critical factors (102). These factors lead to membrane structural damage and ultimately drive the occurrence of ferroptosis.

**Figure 5:**
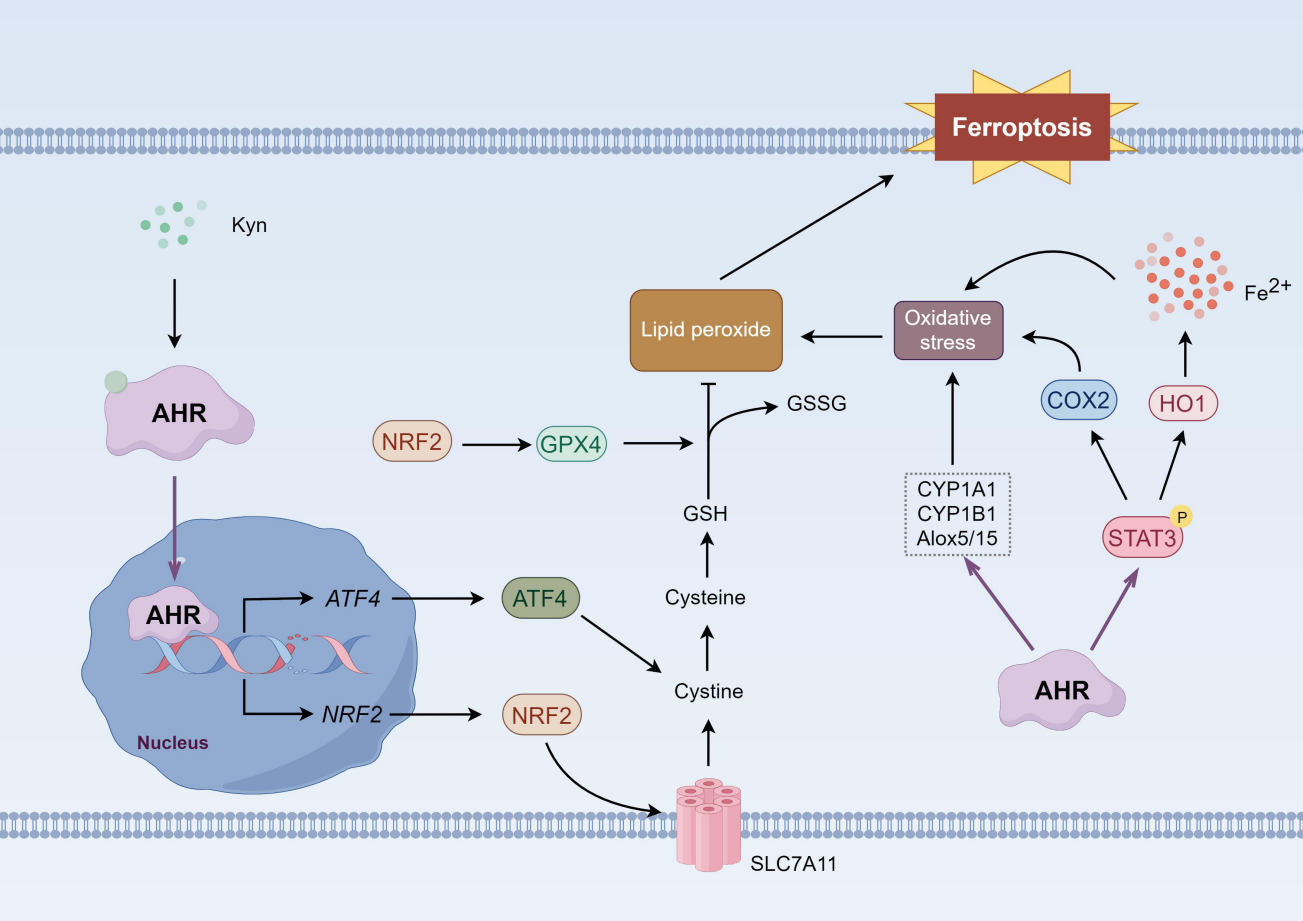
AHR-mediated regulation of ferroptosis pathways. Ferroptosis is a form of PCD characterized by iron-dependent lipid peroxidation. AHR-regulated ferroptosis primarily influences processes related to lipid peroxidation and oxidative stress. Upon binding to ligands such as Kyn, AHR translocates into the nucleus and promotes the transcription of ATF4 and NRF2. ATF4 and NRF2 inhibit lipid peroxidation and ferroptosis by enhancing the reduction of lipid peroxides. Conversely, AHR can also facilitate ROS production and oxidative stress by modulating the expression of enzymes involved in lipid peroxidation, such as CYP1A1, CYP1B1, and Alox5/15, thereby promoting lipid peroxidation. Furthermore, AHR-mediated STAT3 phosphorylation upregulates the expression of COX2 and HO1. These enzymes exacerbate oxidative stress, resulting in lipid peroxide accumulation and the consequent induction of ferroptosis.PCD, programmed cell death; AHR, aryl hydrocarbon receptor; Kyn, kynurenine; ATF4, activating transcription factor 4; NRF2, nuclear factor erythroid 2-related factor 2; ROS, reactive oxygen species; CYP1A1, cytochrome P450 1A1; CYP1B1, cytochrome P450 1B1; COX2, cyclooxygenase 2; HO1, heme oxygenase 1; STAT3, signal transducer and activator of transcription 3.

Although AHR has traditionally been recognized for its roles in xenobiotic metabolism and immune regulation, recent studies have highlighted its critical function in regulating ferroptosis (**Figure 5**). Lysosomal cystine levels serve as a key determinant of cellular sensitivity to ferroptosis, with AHR acting as a central signaling molecule that connects lysosomal cystine status to nuclear stress responses (103). Under conditions of lysosomal cystine deficiency, AHR is activated and promotes the transcriptional expression of its downstream gene activating transcription factor 4 (ATF4), thereby enhancing cystine synthesis and ultimately reducing the susceptibility of tumor cells to ferroptosis (103). Additionally, in lung cancer cells, the endogenous ligand Kyn-activated AHR can directly bind to the XRE in the NRF2 promoter region, thereby promoting the transcription of NRF2 (104). As a key regulator of ferroptosis, NRF2 subsequently upregulates the expression of solute carrier family 7 member 11 (SLC7A11) and glutathione peroxidase 4 (GPX4) (105). This enhances cystine uptake and glutathione (GSH) synthesis, ultimately suppressing ferroptosis (105). However, a study has revealed a contrasting role for AHR activation in a murine liver disease model, where it promotes ferroptosis by enhancing iron deposition and lipid peroxidation (106). This effect is mediated through the AHR-STAT3 axis, which transcriptionally upregulates heme oxygenase 1 (HO1) and cyclooxygenase-2 (COX2) (106). Liu et al. showed that AHR activation triggers selective ferroptosis in HSCs, leading to reduced collagen deposition and ultimately attenuated liver fibrosis in mice (107). Furthermore, as a transcription factor, AHR may exacerbate ferroptosis by upregulating the expression of genes involved in lipid peroxidation (such as CYP1A1, CYP1B1, Alox5 and Alox15), thereby promoting the production of lipid-derived ROS and disrupting cellular redox homeostasis (19, 108, 109). It is important to note that the regulatory role of AHR in ferroptosis is not simply pro- or anti-ferroptotic but exhibits significant dependence on cell type and the disease microenvironment. In cells with elevated NRF2 activity and low basal ROS, AHR−mediated signaling through NRF2/SLC7A11/GPX4 tends to prevail, conferring resistance to ferroptosis. In contrast, cells that are abundant in iron and exhibit active lipid metabolism may undergo AHR-mediated upregulation of CYP1A1, CYP1B1, or ALOX, thereby promoting lipid peroxidation. Furthermore, the same AHR ligand can elicit opposing effects across tissues. This difference forms the foundation for the development of precision therapies for different diseases.

### 3.6 Determinants of AHR functional specificity in PCD regulation

Evidence supports that AHR exerts opposing effects on PCD, either promoting or suppressing cell death depending on stimuli and tissues. These divergent outcomes reflect the ligand, the activated cell type, and the balance between canonical and non-canonical AHR signaling.

AHR ligands differ significantly in their binding affinity, metabolic stability, and the conformational changes they induce in the receptor. High-affinity compounds that resist metabolic clearance, such as TCDD, keep the receptor in the nucleus for extended periods, driving sustained expression of CYP1A1 and related oxidative genes. The resulting accumulation of reactive species and DNA damage often tips the balance toward apoptosis or ferroptosis (61, 110). By contrast, endogenous ligands such as FICZ are rapidly eliminated through CYP1A1-dependent metabolism, resulting in only transient AHR signals that may favor adaptive responses over cell death (111). Kynurenine, however, acts as a precursor that requires conversion into potent derivatives to achieve full agonism (37). Ligand identity thus governs not only signal intensity but also its duration and quality, and these kinetic features determine the eventual outcome as protective or deleterious.

The cell type in which AHR is activated is equally critical. The same ligand can drive opposite PCD responses in different cells, because each cell type has its own transcriptional and metabolic baseline. In intestinal epithelial cells, AHR activation represses necroptosis by down−regulating specific genes (68). In lymphocytes, however, the same AHR stimulus tends to promote pyroptosis (87). Basal redox capacity, the relative AHR/ARNT/AHRR expression, and co-regulator availability all modulate the sensitivity of AHR target genes (7). These intrinsic differences effectively reinterpret the same ligand signal into distinct cell-specific outputs. In addition to the canonical genomic pathway, AHR operates through non-genomic mechanisms, including the AHR-CUL4B ubiquitin ligase axis, the AHR-c-Src cascade, and the AHR-NF-κB interaction, to regulate programmed cell death (PCD) effectors at the post-translational level. The pathway that predominates depends on the ligand structure, cell type, and the local expression of scaffolding or accessory proteins.

These influencing factors are not independent. The characteristics of ligands determine which non-genomic pathways are activated, while the cellular environment alters the interpretation of transcriptional and post-transcriptional signals. The ultimate impact on PCD results from the integration of these input signals.

## 4 AHR-regulated PCD in diseases and therapy

In recent decades, the discovery of numerous endogenous ligands of AHR has significantly expanded the scope of its research, moving far beyond the initial focus on toxicology. Significant advances have been made in understanding the multiple roles of AHR in pathological processes such as inflammatory bowel disease (IBD), sepsis, neurodegenerative disorders, respiratory system diseases, and cancer. AHR contributes critically to disease progression by regulating multiple PCD pathways. Consequently, targeting these relevant mechanisms emerges as a promising therapeutic direction for numerous diseases (**Table 1**).

**Table 1:** Investigations of AHR-regulated PCD for the treatment of multiple human diseases.

Diseases	PCD type	AHR pathological function	Treatment	Target (AHR)	Ref
Immune and inflammatory diseases					
Inflammatory bowel disease	Pyroptosis	Suppressing K+ efflux-mediated macrophage pyroptosis	–	–	(97)
	Necroptosis	Inhibiting the formation of the RIPK1-RIPK3-MLKL complex to alleviate IECs necroptosis	I3C	Promote activation	(72)
	Apoptosis	Promoting autophagy mediated by the mTORC1 pathway to reduce apoptosis of IECs cells	Alpinetin	Promote activation	(112)
	Autophagy	Upregulating the expression of autophagy-related proteins Becn1 and Atg5 to induce autophagy	Astragalus polysaccharide	Promote activation	(113)
Sepsis	Apoptosis	Promoting macrophage apoptosis and enhancing ROS generation	RY103	Inhibit activation	(114)
	Ferroptosis	Inducing the expression of the ferroptosis-related gene Pla2g4a	6,2’,4’-trimethoxyflavone	Inhibit activation	(108)
Osteoarthritis	Pyroptosis	Inducing pyroptosis of chondrocytes mediated by the NF-κB/NLRP3/caspase-1 signaling axis activation	Spermidine	Inhibit activation	(98)
Neurologic diseases					
Traumatic brain injury	Ferroptosis	Promoting lipid peroxidation induced by CYP1B1 and increasing iron accumulation	3-n-butylphthalide	Inhibit activation	(115)
Neurotoxicity	Apoptosis	Inducing endoplasmic reticulum stress to drive neuronal apoptosis	Hesperetin	Inhibit activation	(116)
Cognitive impairment	Pyroptosis	Promoting CYP1A1-mediated neuronal pyroptosis	Urolithin A	Inhibit activation	(117)
	Pyroptosis	Increasing pyroptosis in hippocampal tissue by activating the NFκB pathway	Irisin	Inhibit activation	(118)
Multiple sclerosis	Apoptosis	Promoting death receptor (Fas/FasL) mediated T cell apoptosis	Resveratrol	Promote activation	(119)
Alzheimer’s disease	Pyroptosis	Suppressing NF-κB-mediated NLRP3 inflammasome activation	Indole	Promote activation	(120)
Respiratory system diseases					
Pulmonary hypertension	Apoptosis	Inducing PAECs apoptosis through crosstalk with the NF-κB pathway	Salidroside	Inhibit activation	(121)
Chronic obstructive pulmonary disease	Apoptosis	Promoting CYP1A1-mediated pulmonary cell apoptosis	Ally isothiocyanate	Inhibit activation	(122)
	Apoptosis	Promoting CYP1A1-mediated pulmonary cell apoptosis	Formononetin	Inhibit activation	(123)
Allergic rhinitis	Pyroptosis	Inducing CYP1A1-ROS-NLRP3 pathway activation		–	(95)
Asthma	Ferroptosis	Upregulating the expression of cPLA2 to promote lipid peroxidation in AECs			(124)
Cancer					
Small-cell lung cancer	Apoptosis	Promoting ERK/AKT/FOXM1 signaling pathway activation	Clofazimine	Inhibit activation	(125)
Colorectal cancer	Apoptosis	Promoting CYP1A1-mediated tumor cell apoptosis	BOLD-100	Promote activation	(126)
Breast cancer	Pyroptosis	Suppressing the NLRP3 inflammasome activation by regulating the Nrf2/HO-1 signaling axis	Raloxifene	Promote activation	(127)
Colorectal cancer	Apoptosis	Inducing TNF/TNFR-mediated tumor cell apoptosis	Chrysin	Promote activation	(58)
Lymphoma	Apoptosis	Promoting FasL/FasR- mediated EL4 cell apoptosis	3,3’-diindolylmethane	Promote activation	(128)
Non-small cell lung cancer	Ferroptosis	Promoting NRF2/GPX4 signaling pathway activation	Resveratrol	Promote activation	(129)
Other diseases					
Chronic heart failure	Apoptosis	Promoting MAPK/ERK signaling pathway activation	Resveratrol	Inhibit activation	(86)
Vascular diseases	Apoptosis	Promoting the expression of NRF2 and its downstream antioxidant enzymes in human endothelial cells	Rutaecarpine	Promote activation	(130)
Metabolic dysfunction-associated steatohepatitis (MASH)	Ferroptosis	Increasing aberrant iron overload to trigger ferroptosis	CH223191	Inhibit activation	(131)

### 4.1 Immune and inflammatory diseases

IBD is a chronic inflammatory condition of the gastrointestinal tract characterized by an abnormal immune response (132). It typically manifests with severe gastrointestinal symptoms of unknown cause and has an unpredictable prognosis, posing a significant health challenge worldwide (133). Substantial evidence indicates that AHR plays a pivotal role in maintaining intestinal homeostasis by regulating the differentiation, function, and cellular destiny of key immune cells, including intraepithelial lymphocytes, macrophages, and innate lymphoid cells (134, 135). Conditional deletion of AHRR specifically in intestinal intraepithelial lymphocytes (IELs) triggered a cascade of AHR disinhibition and CYP1A1 upregulation, which promoted lipid peroxide accumulation and ferroptosis (19). In a recent study, Gao et al. further elucidated that AHR activation suppresses macrophage pyroptosis by enhancing polyamine biosynthesis and subsequently inhibiting K+ efflux, thereby alleviating inflammatory injury in the colon (97). This macrophage-intrinsic mechanism underscores AHR’s protective role in limiting inflammasome-mediated tissue damage during intestinal inflammation. Furthermore, as a dietary-source agonist of AHR, I3C activates AHR to induce the ubiquitination and degradation of RIPK1. This process consequently inhibits the assembly of the necrosome (the RIPK1-RIPK3-MLKL complex), thereby mitigating necroptosis in intestinal epithelial cells (IECs) (72). Alpinetin is a flavonoid derived from Chinese herbal medicine that exhibits broad-spectrum anti-inflammatory activity. It has been shown to promote autophagy mediated by the mTORC1 pathway through activation of AHR, which ultimately induces the reduction of apoptosis in IECs (112). Similarly, astragalus polysaccharide (APS), a bioactive component derived from the root of *Astragalus membranaceus*, exerts therapeutic effects in the dextran sulfate sodium (DSS)-induced mouse colitis model by activating AHR to upregulate the expression of autophagy-related proteins (113).

Sepsis is a life-threatening condition characterized by a dysregulated host response to infection, often accompanied by acute organ dysfunction and immune impairment. As previously discussed, AHR can induce multiple forms of cell death in key immune and parenchymal cells, thereby exacerbating the pathological progression of sepsis. During the early phase of sepsis, IDO1 activates the AHR-CYP1A1 signaling pathway by metabolizing tryptophan to produce Kyn. This activation promotes macrophage apoptosis and enhances ROS generation, thereby exacerbating inflammatory responses and contributing to tissue injury (114). In contrast, administration of the IDO1 inhibitor RY103 effectively blocks the IDO1-AHR-CYP1A1 axis, attenuates the cytokine storm, and exerts a protective effect on organ function (114). Furthermore, Cheng et al. demonstrated that in sepsis-associated acute thymic atrophy, activation of the IDO1/Kyn/AHR signaling axis induces mitochondrial damage and lipid peroxidation, thereby promoting ferroptosis and leading to substantial thymocyte loss (108). This cascade ultimately culminates in T cell exhaustion and immune dysfunction, pathological changes that can be effectively suppressed by the AHR inhibitor 6,2’,4’-trimethoxyflavone (TMF) (108). These findings highlight that AHR-driven PCD in immune cells is a crucial mechanism contributing to the dual pathological features of sepsis, which include initial hyperinflammation and subsequent immune paralysis.

Additionally, evidence indicates that the AHR signaling pathway is critically involved in chondrocyte development and growth, and contributes to the pathogenesis of osteoarthritis as well as other cartilage disorders (136). Specifically, research indicates that AHR activation promotes chondrocyte pyroptosis via regulation of the NF-κB pathway (98). The natural compound spermidine, however, can bind to AHR and form a stable complex, effectively suppressing the phosphorylation of IκBα and p65, reducing chondrocyte pyroptosis, and thereby exerting a protective effect against osteoarthritis progression (98).

### 4.2 Neurologic diseases

Targeting AHR-regulated PCDs holds therapeutic promise for various neurological conditions, including traumatic brain injury (TBI), neurotoxicity, multiple sclerosis (MS), and neurodegenerative disorders (137–139).

Neurons in a weight-drop-induced TBI model exhibited a marked increase in AHR expression, suggesting its potential role in the pathology (140). Subsequent research by Yan et al. demonstrated that 3-n-butylphthalide (NBP), a compound derived from celery, effectively mitigated iron accumulation and suppressed neuronal ferroptosis in the brain tissue of TBI mice (115). This neuroprotective effect is at least partially attributable to the inhibition of the AHR-CYP1B1 signaling pathway by NBP (115). Environmental pollutants and dietary toxins are significant risk factors for neurological dysfunction in mammals, and their effects are closely linked to AHR activation (141–143). Aflatoxin B1 (AFB1), a common neurotoxic mycotoxin found in food and feed, activates the AHR and triggers endoplasmic reticulum stress (ER stress) in hippocampal neurons (116). Notably, research indicates that hesperetin mitigates AFB1-induced neurotoxicity by inhibiting the activation of AHR, thereby reducing neuronal apoptosis driven by ES stress (116). Furthermore, the industrial pollutant cadmium (Cd) induces the activation of the NLRP3 inflammasome by activating the AHR-CYP1A1 pathway, thereby triggering neuronal pyroptosis, while urolithin A (UA) can effectively alleviate the neurotoxicity and cognitive impairment caused by Cd in mice by inhibiting this pathway (117). Cognitive impairment is also a frequent complication associated with chronic kidney disease (CKD) (144). It has been reported by Zhang et al. that mice with CKD display deficits in cognitive behavior and a marked increase in hippocampal pyroptosis, which is mechanistically linked to the activation of the AHR-NF-κB pathway (118). Their work further demonstrated that inhibiting this pathway with irisin ameliorates CKD-associated cognitive impairment (118). In contrast, it has been reported that AHR activity in the serum of patients with MS is inversely correlated with the degree of central nervous system atrophy (145). In the experimental autoimmune encephalomyelitis (EAE) model, resveratrol has been shown to promote T cell apoptosis in the spinal cord by activating AHR, thereby alleviating disease progression (119).

Disruption of protein homeostasis is a key pathogenic driver of numerous neurodegenerative diseases. AHR contributes to neuronal proteostasis by promoting the transcriptional expression of neprilysin (NEP). This upregulation of NEP facilitates the clearance of β-amyloid, thereby exerting neuroprotective effects in mouse models of Alzheimer’s disease (AD) (146). The connection between gut microbiota-derived metabolites and neuroinflammation is increasingly recognized as a critical pathway in neurodegenerative diseases, including AD, with the AHR signaling pathway serving as a critical link (147, 148). Specifically, studies have shown that indole, a gut bacterial metabolite, can activate the AHR signaling pathway, thereby suppressing NF-κB-mediated NLRP3 inflammasome activation and mitigating neuroinflammation (120). This immunomodulatory effect is mediated, at least in part, by regulating microglial pyroptosis and the subsequent release of proinflammatory cytokines. Thus, targeting the gut microbiota–AHR–neuroinflammation axis may represent a promising strategy for intervening in neurodegenerative diseases.

### 4.3 Respiratory system diseases

As a core regulator of mucosal barrier function, the expression and functional activity of AHR are critical for maintaining respiratory system homeostasis (149). Research has demonstrated that AHR can interact with NF-κB to induce inflammatory responses, ultimately leading to apoptosis in lung tissue (150). Salidroside (SAL), a natural compound extracted from the roots of *Rhodiola rosea*, has been confirmed to bind to AHR and inhibit its nuclear translocation. This action attenuates oxidative stress and inflammatory responses, consequently reducing apoptosis in pulmonary arterial endothelial cells (PAECs) (121). Similarly, ally isothiocyanate (AITC), an active constituent of *Semen Sinapis Albae*, mitigates the pathological progression of chronic obstructive pulmonary disease (COPD) by suppressing pulmonary cell apoptosis mediated through the AHR-CYP1A1 signaling pathway (122). Moreover, Li et al. ‘s research demonstrated that the isoflavonoid formononetin (FMN) targets the same pathway, markedly inhibiting cigarette smoke (CS)-induced apoptosis in lung tissue, which further emphasizes the significance of the AHR-CYP1A1 axis as a potential therapeutic target for COPD (123).

Exposure to PM_2.5_ is a critical environmental risk factor for various respiratory diseases, with particularly adverse effects on the upper respiratory tract (151, 152). Studies indicate that in patients with allergic rhinitis (AR), PM_2.5_ can disrupt the structure of the nasal epithelial barrier through the NLRP3-mediated pyroptosis pathway (95). Specifically, as an important “environmental sensor” in the body, AHR can be activated by polycyclic aromatic hydrocarbons (PAHs) present in PM_2.5_. This activation subsequently triggers a cascade reaction through the downstream CYP1A1-ROS-NLRP3 pathway, ultimately inducing pyroptosis in nasal mucosal epithelial cells (95). Moreover, indeno[1,2,3-cd]pyrene (IP), a major PAH component in PM_2.5_, can specifically bind to AHR (153). This binding event drives the upregulation of cytosolic phospholipase A2 (cPLA2) at the transcriptional level, which accelerates lipid peroxidation in airway epithelial cells (AECs) and results in ferroptosis (124).

### 4.4 Cancer

Carcinogenesis is typically characterized by unlimited cell proliferation and the evasion of regulated cell death (154, 155). Although alterations in various modes of PCD represent hallmarks of cancer development, their functional impacts on tumor cells are distinct (156). Generally, cell death participates in tumor progression through two principal mechanisms: 1) Under stress conditions, the initiation of cell death leads to organelle degradation and disruption of cellular architecture, which helps maintain homeostasis and suppresses cancer cell proliferation; 2) Certain modes of cell death can trigger persistent tissue inflammation, which in turn promotes tumorigenesis and growth (156).

AHR exhibits widespread and elevated expression across various human cancers. Nevertheless, studies have revealed that it plays a dual regulatory role in tumor progression, with its ultimate biological effects being highly context-dependent and influenced by factors such as the specific tumor type and the tumor microenvironment (157). Activated AHR enhances the proliferation, migration, and colony-forming ability of small-cell lung cancer (SCLC) cells through the upregulation of the oncogenic ERK/AKT/FOXM1 signaling pathway, which consequently increases tumor invasiveness. Subsequent studies found that the anti-leprosy drug clofazimine (CLF) competitively binds to the ligand-binding domain of AHR, suppressing its transcriptional activity and effectively inhibiting the malignant phenotype of SCLC cells while inducing apoptosis (125). However, in colorectal cancer, the ruthenium drug BOLD-100 induces tumor cell apoptosis by activating the AHR/CYP1A1/ROS signaling axis (126). Furthermore, raloxifene, a second-generation selective estrogen receptor modulator, is clinically used for the prevention of invasive breast cancer in postmenopausal women (158). Recent research from Professor Park’s team has revealed that this agent exerts anti-tumor effects in breast cancer by suppressing the NLRP3 inflammasome activation. The underlying mechanism involves the upregulation of aryl hydrocarbon receptor expression, thereby activating the downstream NRF2/HO-1 signaling axis and ultimately reducing intracellular reactive oxygen species levels (127).

Notably, numerous plant-derived compounds have demonstrated promising effects in cancer prevention and control in recent years (159, 160). These compounds can induce tumor cell death by targeting intracellular regulatory factors, such as AHR (161). For example, the flavonoid chrysin induces TNF-TNFR-mediated apoptosis in human colorectal cancer cells by activating the AHR signaling pathway, and this pro-apoptotic effect was blocked by AHR knockdown using shRNA (58). Similarly, resveratrol, a polyphenolic compound widely found in plants, has been reported to suppress the growth and proliferation of various cancer cells, including those from lung, gastric, and liver cancers (58). Singh et al.’s research demonstrated that resveratrol induces apoptosis in lymphoma EL4 cells by activating AHR to initiate the FASL–FASR signaling axis (128). Additionally, recent research indicates that through the modulation of the AHR/NRF2/GPX4 signaling pathway, the cruciferous vegetable-derived compound DIM can trigger ferroptosis in non-small cell lung cancer cells (129). Despite the considerable potential in this field, current investigations into AHR remain largely limited to specific tissue contexts. In the tumor microenvironment, AHR-mediated PCD regulation takes on additional complexity due to its impact on both tumor cells and infiltrating immune cells. For instance, AHR-driven apoptosis in cytotoxic T lymphocytes can impair anti-tumor immunity, whereas ferroptosis in regulatory immune populations may reshape the tumor immune landscape in ways that either promote or restrain tumor progression. Therefore, it is necessary to systematically elucidate the differential roles of AHR in various tumor types and immune cell subsets in the future. This will provide a basis for designing targeted cancer treatments that focus on AHR, with the aim of inhibiting tumors without damaging protective anti-tumor immunity.

### 4.5 Other diseases

Chronic kidney disease (CKD) significantly increases the risk of cardiovascular events, including chronic heart failure, in patients (162). The accumulation of uremic toxins, such as KYN, during the progression of chronic kidney disease (CKD) serves as an important link between renal dysfunction and the occurrence of cardiovascular injury (163). Recent studies indicate that KYN promotes cardiomyocyte apoptosis by activating the AHR/MAPK/ERK signaling pathway, while resveratrol effectively suppresses this process. This finding suggests a potential adjunctive therapeutic strategy for cardiovascular protection in patients with CKD (86). However, the activation of AHR by rutaecarpine (RUT) significantly upregulates the expression of NRF2 and its downstream antioxidant enzymes in human endothelial cells, thereby suppressing oxidative stress-induced apoptosis (130). AHR is also an important regulatory factor in the development of liver diseases (164). Studies have shown that AHR is expressed at abnormally high levels in the liver tissues of metabolic dysfunction-associated steatotic liver disease (MASLD) (131). By modulating the PTEN/AKT/β-catenin signaling pathway, AHR exacerbates intracellular iron overload in hepatocytes, promoting the ferroptosis process and ultimately leading to the aggravation of liver histopathological injury. Both *in vivo* and *in vitro* experiments indicate that specifically inhibiting AHR, for example, using the antagonist CH223191, can effectively reduce hepatic lipid deposition and suppress iron overload and ferroptosis, offering a potential strategy for ameliorating MASLD (131). In summary, while targeting AHR-regulated PCD has attracted considerable attention, its effects are context-dependent and vary based on ligands and disease states. This necessitates further exploration of the detailed mechanisms involved.

## 5 Conclusions and future directions

PCD plays a critical role in organismal homeostasis and the clearance of abnormal cells, and its dysregulation is a major driver in the development and progression of various diseases. AHR, a ligand-activated transcription factor, has been increasingly recognized for its pivotal role in modulating cellular homeostasis and PCD. Accumulating evidence indicates that AHR can directly regulate the expression of genes associated with various PCD modalities such as apoptosis, autophagic cell death, pyroptosis, and ferroptosis through transcriptional mechanisms. Furthermore, AHR also acts as a critical signaling hub, regulating key biological processes including oxidative stress and inflammatory responses, thereby indirectly modulating cell death pathways. Importantly, the immunological functions of AHR, which encompass its effects on immune cell survival, differentiation, and effector activity, are closely linked to its regulation of PCD. By controlling the death and persistence of immune cells, AHR profoundly influences the initiation, amplification, and resolution of inflammatory responses across various disease contexts.

Notably, both the overactivation and functional inhibition of AHR may lead to an imbalance in PCD, though the specific effects depend on the pathological context. For instance, in a chronic inflammatory state, sustained AHR signaling may promote inflammasome activation or lipid peroxidation, thereby driving pyroptosis or ferroptosis and accelerating tissue damage. This is particularly relevant in immune cells such as macrophages and T lymphocytes, where AHR-driven PCD can either limit or exacerbate tissue inflammation depending on the local milieu. In the tumor microenvironment, however, AHR may facilitate tumor survival and immune escape by upregulating anti-apoptotic factors in tumor cells while simultaneously inducing immunosuppressive effects, including cell death or functional impairment, in infiltrating lymphocytes. Furthermore, studies suggest that AHR closely interacts with metabolic enzymes, such as cytochrome P450 family members, and key signaling proteins, including NF-κB and the antioxidant transcription factor NRF2. These findings may offer novel targets for combined therapeutic interventions.

While targeting AHR-regulated PCD has shown promise in preclinical models, the clinical translation remains preliminary and requires careful contextual evaluation. Several key questions still need to be addressed. For instance, why does AHR exert distinct functions across different tissues or cell types? How does AHR, within a complex pathological microenvironment, recognize specific signals and guide the choice of PCD modality? Therefore, future research needs to systematically reveal the specific molecular mechanisms by which AHR regulates PCD in various disease contexts, with particular attention to its roles in different cell types and pathological stages. Building on this, the development of highly selective AHR modulators and exploring their combined use with existing therapeutic strategies are expected to provide potential therapeutic directions for the prevention and treatment of relevant diseases.

## Glossary

AHR: aryl hydrocarbon receptor
AHRR: AHR repressor
AIP: AHR interacting protein
AKT: protein kinase B
AMPK: AMP-activated protein kinase
APS: astragalus polysaccharide
ARNT: AHR nuclear translocator
ATF4: transcription factor 4
ATG5: autophagy related 5
B[a]P: benzo[a]pyrene
Bax: Bcl-2-associated X protein
bHLH: basic helix-loop-helix
Bid: BH3-interacting domain death agonist
β-NF: β-naphthoflavone
ceRNA: competitive endogenous RNA
COX2: cyclooxygenase-2
cPLA2: cytosolic phospholipase A2
CUL4B: cullin 4B
CYP1A1: cytochrome P450 1A1
DAMPs: damage-associated molecular patterns
DIM: 3,3’-diindolylmethane
DSS: dextran sulfate sodium
eEF2K: eukaryotic elongation factor 2 kinase
ESR: estrogen receptor
FasL: Fas ligand
FICZ: 6-formylindolo[3,2-b]carbazole
GPX4: glutathione peroxidase 4
GSDMD: gasdermin D
HO1: heme oxygenase 1
HSP90: heat shock protein 90
I3C: indole-3-carbinol
I3P: indole-3-pyruvate
IAP: inhibitor of apoptosis protein
IBD: inflammatory bowel disease
IDO1: indoleamine 2,3-dioxygenase 1
IECs: intestinal epithelial cells
IELs: intestinal intraepithelial lymphocytes
KMO: kynurenine monooxygenase
Kyn: kynurenine
LC3: light chain 3
LPS: lipopolysaccharide
LXA₄: lipoxin A₄
MAPK: mitogen-activated protein kinase
MLKL: mixed lineage kinase domain-like protein
MOMP: mitochondrial outer membrane permeabilization
mTOR: mammalian target of rapamycin
NF-κB: nuclear factor-κB
NLRP3: NOD-like receptor (NLR) family pyrin domain-containing 3
NRF2: nuclear factor erythroid 2-related factor 2
PAS: Per-Arnt-Sim
PCD: programmed cell death
PUFAs: polyunsaturated fatty acids
RhoA: Ras homolog gene family member A
RIPK: receptor-interacting protein kinase
ROS: reactive oxygen species
SLC7A11: solute carrier family 7 member 11
SOCS2: suppressor of cytokine signaling 2
STAT3: signal transducer and activator of transcription 3
TCDD: 2,3,7,8-tetrachlorodibenzo-p-dioxin
TEACOPs: trace extended aromatic condensation products
TLR: Toll-like receptor
TNF-α: tumor necrosis factor-α
XAP2: X-associated protein 2
XIAP: X-linked inhibitor of apoptosis
XREs: xenobiotic response elements
